# Breakdown Characteristics of GaN DMISFETs Fabricated via Mg, Si and N Triple Ion Implantation

**DOI:** 10.3390/mi15010147

**Published:** 2024-01-18

**Authors:** Tohru Nakamura, Michitaka Yoshino, Toru Toyabe, Akira Yasuda

**Affiliations:** 1 Research Center for Micro-Nano Technology, Hosei University, Tokyo 184-0003, Japan; michitaka.yoshino.86@hosei.ac.jp; 2 Faculty of Engineering, Toyo University, Saitama 350-8585, Japan; 3 Department of Electrical and Electronic Engineering, Faculty of Science and Engineering, Hosei University, Tokyo 184-8584, Japan

**Keywords:** GaN, ion implantation, MISFET, Mg, Si, CAVET

## Abstract

Mg-ion-implanted layers in a GaN substrate after annealing were investigated. Implanted Mg atoms precipitated along the edges of crystal defects were observed using 3D-APT. The breakdown characteristics of a GaN double-diffused vertical MISFET (DMISFET) fabricated via triple ion implantation are presented. A DMISFET with Si-ion-implanted source regions was formed in Mg-ion-implanted p-base regions, which were isolated from adjacent devices by N-ion-implanted edge termination regions. A threshold voltage of −0.5 V was obtained at a drain voltage of 0.5 V for the fabricated vertical MISFET with an estimated Mg surface concentration of 5 × 10^18^ cm^−3^. The maximum drain current and maximum transconductance in a saturation region of V_ds_ = 100 V were 2.8 mA/mm and 0.5 mS/mm at a gate voltage of 15 V, respectively. The breakdown voltage in the off-state was 417 V. The breakdown points were determined by the boundary regions between the N- and Mg-implanted regions. By improving heat annealing methods, ion-implanted GaN DMISFETs can be a promising candidate for future high-voltage and high-power applications.

## 1. Introduction

As ion implantation technology can control the number of impurity atoms in a semiconductor material by controlling the current value, it has become indispensable for semiconductor manufacturing, especially for Si ultrafine integrated circuits. Impurity doping via ion implantation requires a heat treatment technique to recover the crystal defects caused by the range of implanted atoms. When ion implantation is performed in silicon, the typical annealing temperature is below 1200 °C, and silicon crystals remain stable within those temperatures. However, in compound semiconductors such as GaAs, ion implantation is not widely used for device manufacturing, and, instead, a method of doping impurity atoms during the epitaxial growth of crystals is usually used. Therefore, it is difficult to manufacture devices with complex structures because the device is completed by growing one layer by one layer in a planar manner. One of the reasons why ion implantation is not used in the manufacture of compound semiconductor devices is that the crystal structure decomposes at the annealing temperature required for crystal recovery [1]. In the wide-bandgap semiconductor GaN, which has attracted attention in recent years, n-type dopant Si is activated at a relatively low temperature [2,3]. It has been difficult to form a p-type layer using Mg, which is a p-type dopant [2,3], due to its high-temperature annealing process and the influence of H, but reports of Mg-doped p-type layers have increased in recent years [4,5,6,7,8,9,10]. In addition, GaN has better physical properties for power devices than SiC [11,12,13,14] and could be useful in a wide range of power switching applications by enabling vertical device structures similar to Si and SiC [15,16,17,18,19,20,21,22]. Compared to lateral devices, vertical devices have the advantage of combining both the high breakdown voltage and low specific on-resistance of the drift region with majority carriers [19,23]. There have been several reports about vertical devices fabricated in Mg-ion-implanted layers in recent years [7,8,9,24]. The authors have already made it possible to form a p-type layer by implanting Mg ions, and they have reported on Metal Oxide Semiconductor FETs (MOSFETs) using ion implantation [9,25]. Many defects were still present in the Mg- and Si-implanted layers after high-temperature annealing, as observed in TEM images [25]. However, the crystallinity of the Mg-doped p-type layer has not yet been elucidated in detail. We have observed the locations of Mg atoms in the ion-implanted layer using Three-Dimensional Atom Probe Tomography (3D-APT) [26,27] and confirmed the crystallinity and non-uniformity of Mg doping after heat treatment.

This report describes the crystal defects in the Mg-ion-implanted p-type layer and reports GaN double-diffused vertical Metal Insulator Semiconductor FETs (DMISFETs) with a termination region using the nitrogen as-implanted layer formed within the layer. The device structure, whose surface is flattened by nitrogen ion implantation, will enable us to fabricate miniaturized devices with complex wiring.

## 2. Mg-Ion-Implanted P-Type Layer

Epitaxial growth technology is widely used for fabricating p-type layers in GaN devices. But it is difficult to make sophisticated structures with only epitaxial growth technology. Though ion implantation is the most promising technology for Si or SiC device fabrication, there are not many reports on GaN device fabrication. One of the reasons for this is the difficulty in forming Mg-doped p-type layers via ion implantation. To fabricate GaN MISFETs, a p-type layer as a dopant of Mg has to be formed. We have already succeeded in fabricating a Mg-doped p-type layer via ion implantation, but we have not yet published detailed results regarding the behavior of Mg atoms in the layer after heat treatment.

Mg ions were implanted through a 50 nm SiNx film into free-standing c-plane (0001) GaN substrates. After Mg ion implantation was performed at an energy of 150 keV with a dose of 5 × 10^14^ cm^−2^, high-temperature annealing at 1230 °C for 1 min in N_2_ gas ambient was carried out. These ion implantation conditions are similar to those used for fabricating the Mg-doped p-base region of the device described in Section 3.

**Figure 1:**
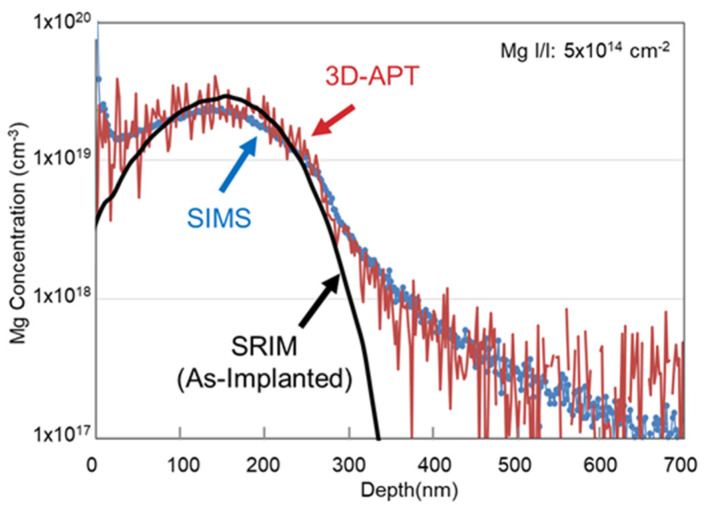
Mg concentration measured using SIMS and 3D-APT.

The implanted Mg profiles in the free-standing GaN substrate measured using Secondary Ion Mass Spectrometry (SIMS) and 3D-APT are shown in Figure 1. 

**Figure 2:**
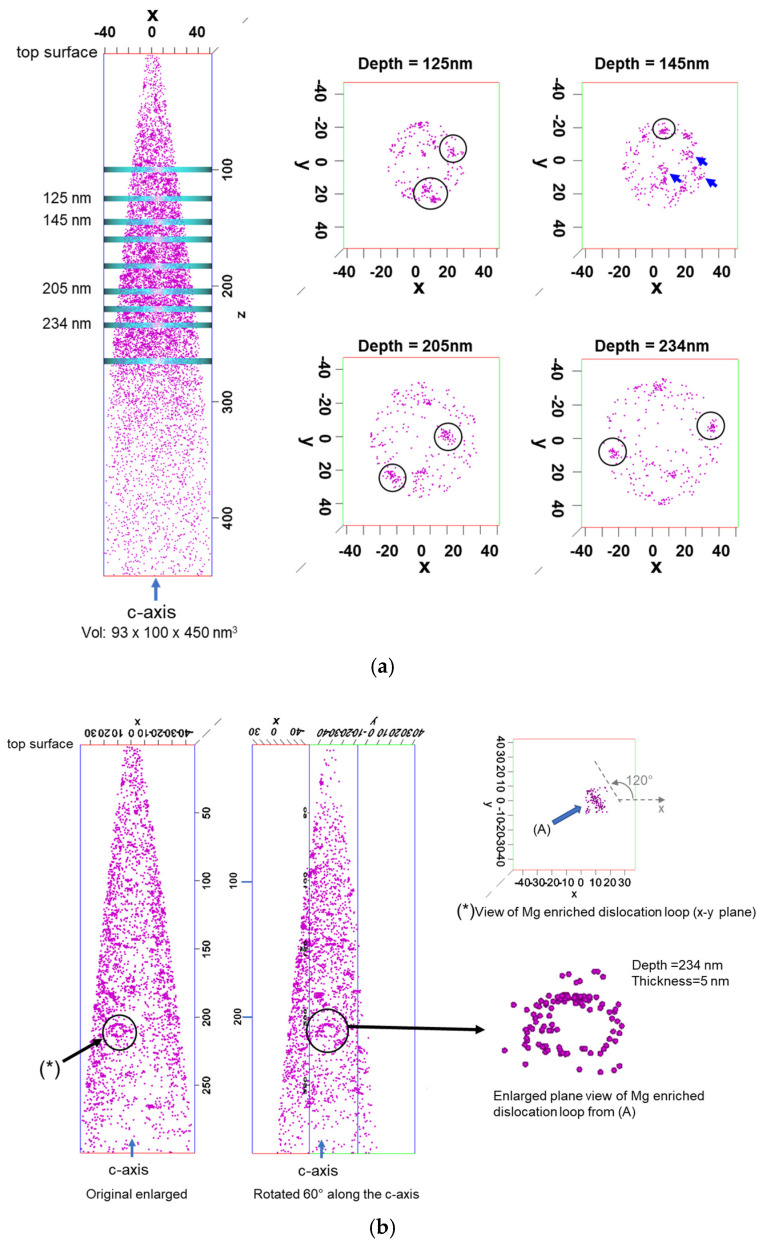
(**a**) Mg atom distribution measured using needle-shaped specimen and plane views of the Mg atoms at different depths in ~5 nm thick slices. (**b**) Mg-enriched dislocation loop at a depth of 230 nm.

A simulated distribution of the as-implanted Mg using ‘The Stopping and Range of Ions in Matter (SRIM-2013)’ [28] software is also shown. Mg atoms slightly updiffused after annealing because of the concentration gradient, and plenty of Ga vacancies were induced by the implantation path [29]. The background of the Si concentration included in the free-standing GaN substrate was about 2 × 10^18^ cm^−3^. Though the as-implanted Mg distribution was considered to be uniform, the Mg concentration derived from 3D-APT was uneven and fluctuated drastically in the depth direction. On the contrary, that derived from SIMS showed a uniform and average profile. This means that the Mg atoms were unevenly doped and not spatially homogeneous, as the analyzed region of 3D-APT was less than several nm square. Figure 2a shows the Mg atom distribution measured using a needle-shaped specimen and plane views of the Mg atoms at different depths in ~5 nm thick slices. 

The Mg distribution was confirmed not to be uniformly distributed laterally within 5 nm depth steps. Most of the Mg-enriched features were identified as clusters, shown as circles in the figure, while at the depth with the maximum Mg concentration, i.e., ~145 nm, a few Mg-enriched features were close to a loop shape (as shown by blue arrows). A more detailed analysis was carried out by rotating 60° along the c-axis of the graph of the Mg atom distribution measured using a needle-shaped specimen, as shown in Figure 2b. The Mg distribution at a depth of 234 nm showed a Mg-enriched dislocation loop parallel to the c-axis but not parallel to either the x-c or y-c plane. The estimated angle between the loop plane and the x-c plane was found to be about 60°.

In the Mg-doped p-type layer implanted at an energy of 150 keV with a dose of 5 × 10^14^ cm^−2^, both Mg-enriched loop and point-like defects were observed. However, the point-like defects were dominant in the analyzed region. The observed loop-like defects were parallel to the c-axis (perpendicular to the original GaN surface). Further improvements in thermal annealing methods are required to form uniformly doped Mg-ion-implanted layers with good crystallinity.

## 3. Device Structure and Fabrication

**Figure 3:**
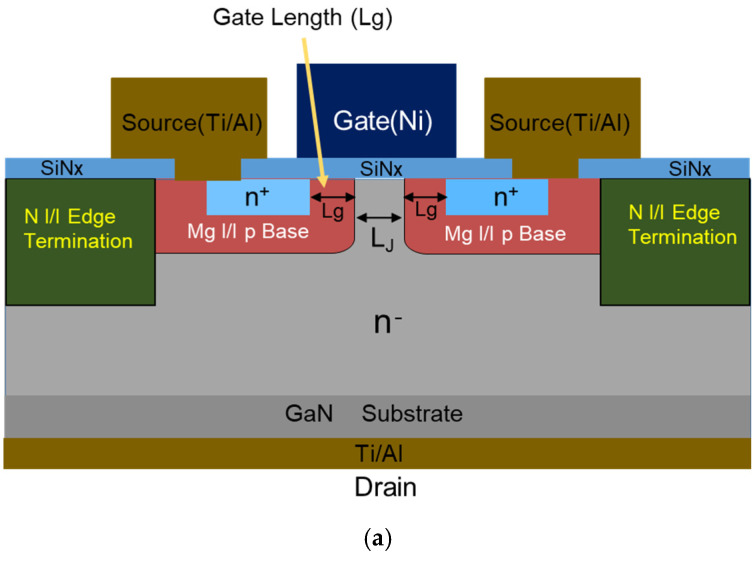

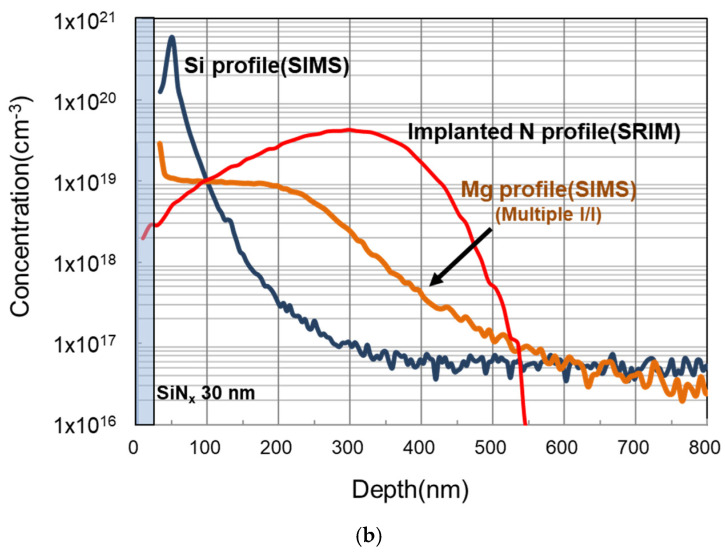
(**a**) Schematic cross-section of the triple-ion-implanted vertical GaN DMISFET. (**b**) Ion-implanted Mg, Si and N profiles.

A schematic cross-section of the ion-implanted GaN DMISFET on the free-standing GaN substrate is shown in Figure 3a. The device structure resembles GaN or Ga_2_O_3_ CAVETs [7,19], but the fabrication process was different. Only ion implantation was used for the doping process, and the device structure was similar to that of SiC or Si DMOSFETs. Channel regions were fabricated in Mg-implanted layers with a tilt angle of 30°, and the gate length was defined in a self-aligned manner by the difference in the lateral range between Mg and Si under the SiN_x_ gate insulator.

The fabrication process of the DMISFET was almost the same as in a previous paper [25], but it was different in some important points, as described below. SiN_x_ gate dielectric films of a 50 nm thickness were sputtered in N_2_ gas ambient. Edge termination regions were also formed via the ion implantation of N at an energy of 100 keV and a dose of 1.0 × 10^15^ cm^−2^ [30]. The device structure has a flattened surface with N-ion-implanted termination compared to the etched one [31], making it possible to create small devices with complex wiring. The implanted Mg and Si profiles measured using SIMS after annealing are shown in Figure 3b. Substituted Ga atoms or damage layers of about 0.1% were produced by N implantation, corresponding to a maximum implanted N concentration of 5 × 10^19^ cm^−3^. Then, the leakage current between the adjacent n-type regions above 2 μm in a p-type layer was suppressed to less than 1 μA/mm. The depth of N-implanted termination was estimated to be 0.4 μm. The Mg surface concentration in the DMISFET channel regions was also estimated to be 5.0 × 10^18^ cm^−3^.

## 4. Device Performance 

The sheet and contact resistances of the ion-implanted source regions were measured using a TLM structure. A low sheet resistance of 139 Ω/Υ and a contact resistance as low as 0.53 Ω mm were obtained [32]. Ohmic contact to the surface of the Mg-ion-implanted regions could not be formed because the carrier concentration of the Mg-ion-implanted contact layer was estimated to be below 1 × 10^18^ cm^−3^ due to a Mg acceptor level as deep as 200 meV [33]. Therefore, it was considered that Mg-ion-implanted p-base regions were maintained at a floating potential or connected as a Schottky contact to the source electrodes.

**Figure 4:**
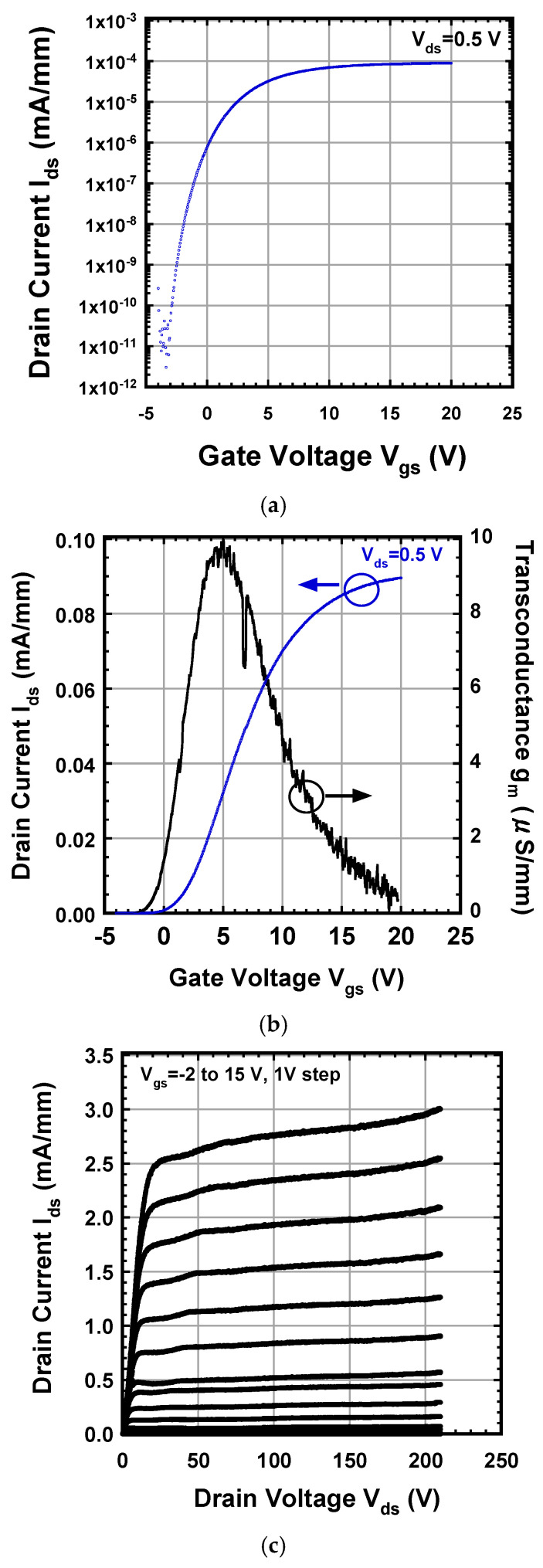

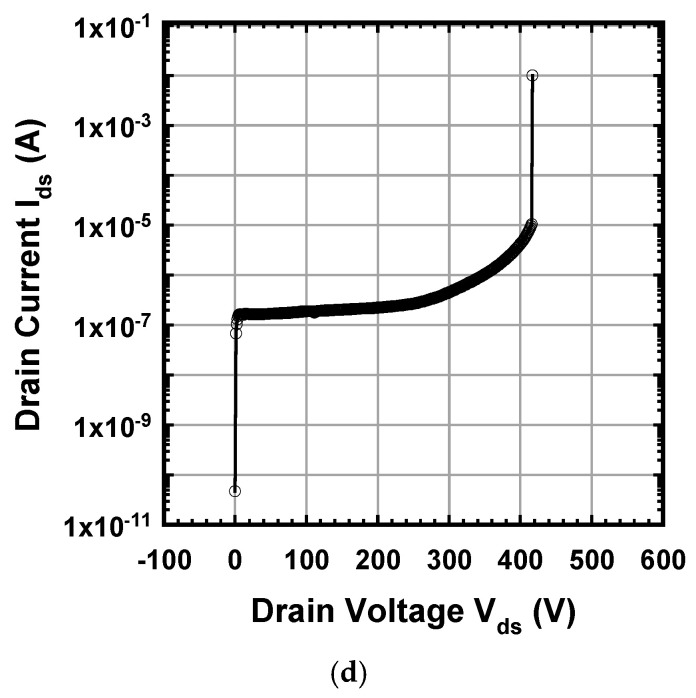
(**a**) Subthreshold characteristics of the DMISFET. (**b**) I_ds_-V_gs_ and g_m_-V_gs_ characteristics of the DMISFET. (**c**) Pulsed I_ds_-V_ds_ characteristics of the DMISFET. (**d**) Off-state I_ds_-V_ds_ characteristics of the DMISFET. The breakdown voltage in off-state was 417 V.

The subthreshold characteristics of the device at a drain voltage of 0.5 V are shown in Figure 4a. I_ds_-V_gs_ and g_m_-V_gs_ characteristics of the fabricated GaN DMISFET at a drain voltage of 0.5 V are shown in Figure 4b. The V_th_ of the DMISFET obtained from the extrapolation of g_m_-V_gs_ characteristics using the extrapolation in the linear region (ELR) method was about −0.5 V.

The pulsed I_ds_-V_ds_ characteristics of the DMISFET by sweep of the V_gs_ value from −2 V to 15 V are shown in Figure 4c. I_dsm_ and g_mmax_ in the saturation region, at a V_ds_ of 100 V, were 2.8 mA/mm and 0.5 mS/mm. I_dsm_ and g_mmax_ were low compared to those in other reports [25,34] because the channel regions under the gate insulators included crystal defects. The reason why I_d_ and g_m_ increased in the saturation region when the drain voltages were over 150 V is considered to be as follows: In the DMISFET structure in which a channel region with a gate length of 0.4 μm [25] is formed by the lateral double diffusion of Mg and Si, the drain current increases due to Drain-Induced Barrier Lowering (DIBL) [35]. Forming a highly concentrated p-base region reduces the increase in drain conductance and increases the threshold; however, it also lowers Ids and gm as a disadvantage. The off-state I_ds_-V_ds_ characteristics of the fabricated GaN DMISFET are shown in Figure 4d. The breakdown voltage was 417 V, which was lower than the expected value for an epitaxial layer thickness of 5 μm.

## 5. Breakdown Characteristics and Discussion

**Figure 5:**
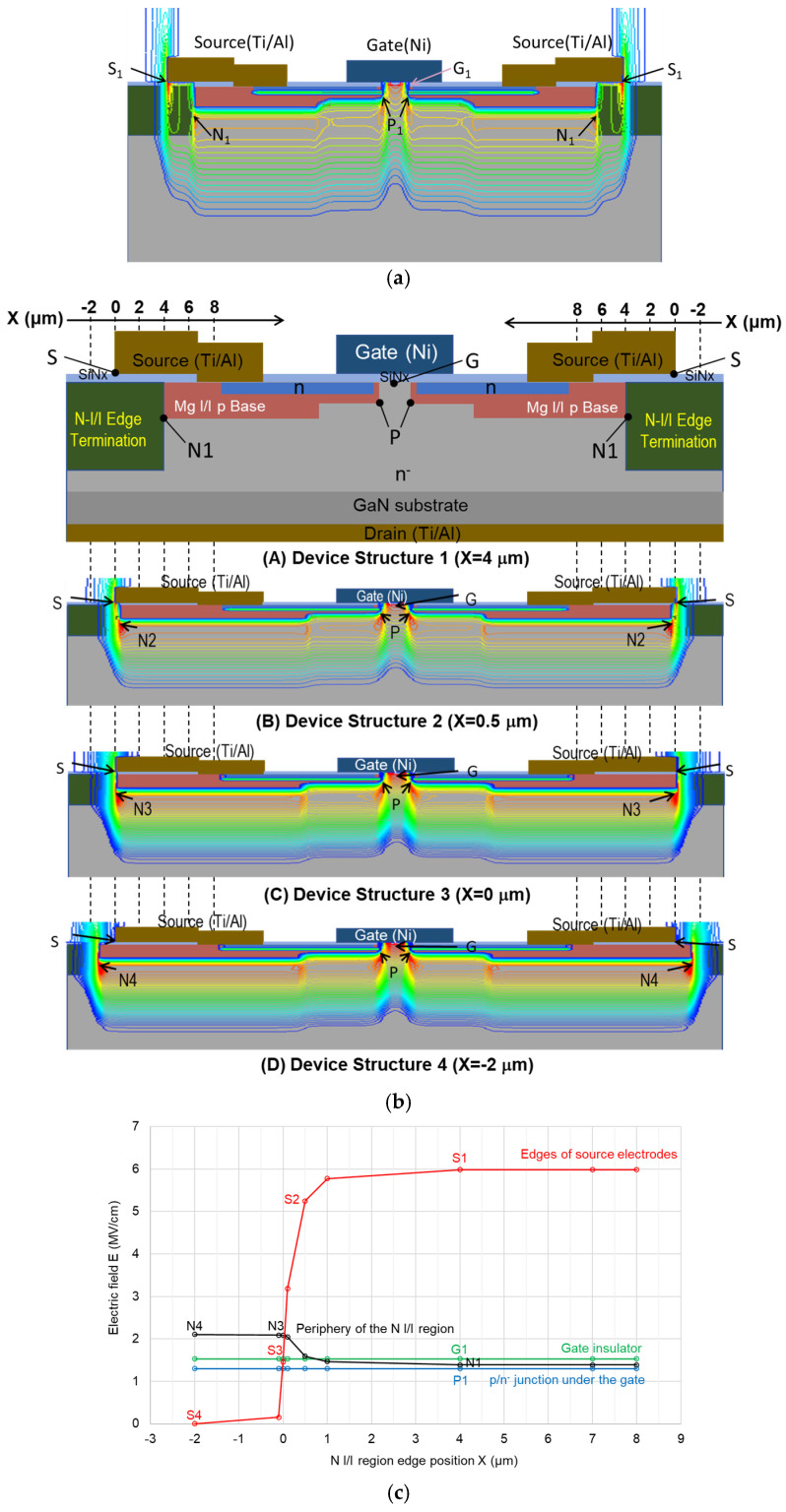

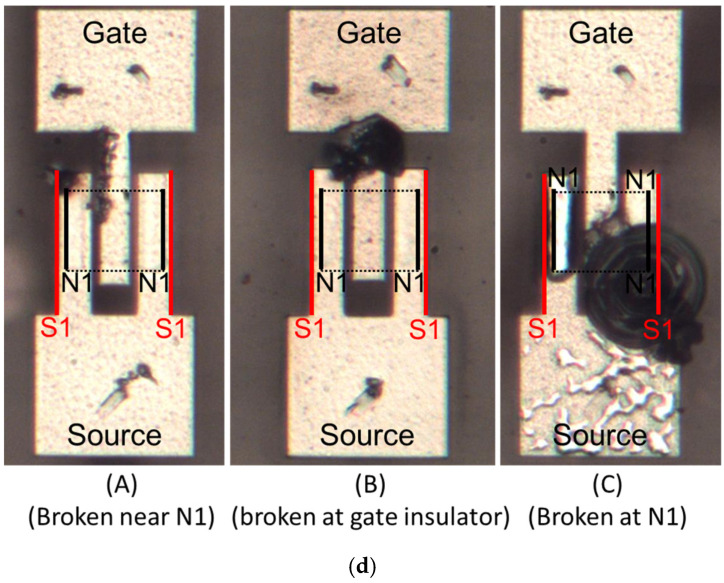
(**a**) Simulated contours of electric field strength at V_ds_ = 100 V. (**b**) Schematic cross-section of the device structures for simulation. The electric field’s highly concentrated positions (G, P, N, S) are also indicated. (**c**) Simulated electric fields at locations where electric field is concentrated. (**d**) Typical examples of the devices after applying a voltage higher than the breakdown. Broken points N and S, relevant to the simulations in (**b**), are shown.

To find the breakdown points in the device, a 2D simulation was carried out to investigate the electric field inside the device. The N I/I termination region was calculated as an insulator with a dielectric constant of 8.9. Simulated contours with an electric field strength of V_ds_ = 100 V are shown in Figure 5a, where the contours with high electric fields are illustrated in red and those with low electric fields are illustrated in blue. The electric field is low in the n- epitaxial region and highly concentrated in the gate insulator (G_1_), at the p/n^-^ junction under the gate (P_1_), at the periphery of the N-ion-implanted (N I/I) region under the Mg-implanted layer (N_1_) and in the insulator regions including the N I/I region under the edges of the source electrodes (S1). To clarify the influence of the N I/I termination regions on the electric field, a simulation was conducted for the devices with different positions of the N I/I region, as shown in Figure 5b. Device Structure 1 is similar to the fabricated device. The N I/I region edge of this structure is located at X = 4 μm and overlaps the source electrodes. The N I/I region edges of Device Structures 2 and 3 are located at X = 0.5 μm and 0 μm. The N I/I region edge of Device Structure 4 is located at X = −2 μm; i.e., the edges of the source electrodes are in the Mg-implanted p-base regions. The drain and GaN substrate layers are not shown in Device Structures 2–4 for simplicity. The simulated contours of the electric field strength at V_ds_ = 100 V are also shown. It is considered that the electric field strength is higher at the end of the source electrode and at the periphery of the N I/I region than in other locations.

The simulated electric fields at V_ds_ = 100 V for the devices with N I/I region edges from X = −2 μm to 8 μm are shown in Figure 5c. At points G and P, the electric fields are constant and do not change, even if N I/I layer edge position X changes. When position X is between 0 μm and 1 μm, the electric fields at the source contact edge, S, increase rapidly, while the electric fields at the N I/I layer edge, N, decrease. If the periphery of the N I/I region under the Mg-implanted layer, N, is located outside the source contact edge (X < 0 μm), the electric field at point S will be the lowest, and the electric field at point N will be the highest. This structure corresponds to the unit cell of the multi-finger power transistor. For Device Structure 1 (X = 4 μm), the breakdown point is estimated to be the source contact edges, as S1 is equal to 6 MV/cm. Figure 5d shows typical device examples after applying a voltage higher than the breakdown. The broken and solid lines in black indicate the boundary between the Mg-implanted p-base region and the N I/I region, N1, and the lines in red indicate the edges of the source electrodes, S1. It is clear that breakdown points occur near the end of the source electrode or near the boundary between the N I/I region and the Mg-implanted p-base region. Although the electric field at point N1 is not high in the simulated results shown in Figure 5a,c, those devices seem to be broken around point N1. This means that the breakdown voltage is determined by the boundary region between the damaged regions produced by N ion implantation and the Mg-implanted regions with Mg-enriched dislocation loops.

## 6. Conclusions

A Mg-ion-implanted p-type layer was investigated using 3D Atomic Prove Tomography. In the Mg-doped layer implanted at an energy of 150 keV with a dose of 5 × 10^14^ cm^−2^, both Mg-enriched loop and point-like defects were observed. The observed loop-like defects were parallel to the c-axis. We also demonstrated a self-aligned GaN DMISFET fabricated via the triple ion implantation of Mg, Si and N. V_th_ obtained from the extrapolation of a linear portion of g_m_ was about −0.5 V. I_dsm_ and g_mmax_ at a drain voltage of 100 V for the DMISFET was 2.8 mA/mm and 0.5 mS/mm, respectively. The breakdown voltage in the off-state was 417 V. The breakdown voltage seemed to be determined by the boundary region between the Mg-doped p-type regions with defects and the N-implanted termination regions. High-performance vertical GaN DMISFETs can be achieved by further improving the sophisticated ion implantation procedure, especially the development of the thermal annealing process.

## Data Availability

Data are contained within the article.
